# Comprehensive characterization of cell-free tumor DNA in plasma and urine of patients with renal tumors

**DOI:** 10.1186/s13073-020-00723-8

**Published:** 2020-02-28

**Authors:** Christopher G. Smith, Tina Moser, Florent Mouliere, Johanna Field-Rayner, Matthew Eldridge, Anja L. Riediger, Dineika Chandrananda, Katrin Heider, Jonathan C. M. Wan, Anne Y. Warren, James Morris, Irena Hudecova, Wendy N. Cooper, Thomas J. Mitchell, Davina Gale, Andrea Ruiz-Valdepenas, Tobias Klatte, Stephan Ursprung, Evis Sala, Antony C. P. Riddick, Tevita F. Aho, James N. Armitage, Samantha Perakis, Martin Pichler, Maximilian Seles, Gabriel Wcislo, Sarah J. Welsh, Athena Matakidou, Tim Eisen, Charles E. Massie, Nitzan Rosenfeld, Ellen Heitzer, Grant D. Stewart

**Affiliations:** 1 Cancer Research UK Cambridge Institute, Li Ka Shing Centre, Robinson Way, Cambridge, CB2 0RE UK; 2 Cancer Research UK Major Centre – Cambridge, Cancer Research UK Cambridge Institute, Li Ka Shing Centre, Robinson Way, Cambridge, CB2 0RE UK; 3 Medical University of Graz, Diagnostic and Research Center for Molecular Biomedicine, Institute of Human Genetics, Graz, Austria; 4 Department of Pathology, Cancer Center Amsterdam, Amsterdam UMC, Vrije Universiteit Amsterdam, De Boelelaan 1117, 1081 HV Amsterdam, The Netherlands; 5 Cambridge Urology Translational Research and Clinical Trials Office, University of Cambridge, Cambridge, CB2 0QQ UK; 6 Cambridge University Hospitals NHS Foundation Trust, Cambridge, CB2 0QQ UK; 7 Wellcome Sanger Institute, Hinxton, CB10 1SA UK; 8 Department of Surgery, University of Cambridge, Cambridge, CB2 0QQ UK; 9 Department of Urology, Royal Bournemouth Hospital, Bournemouth, UK; 10 Department of Radiology, University of Cambridge, Cambridge, CB2 0QQ UK; 11 Department of Internal Medicine Graz, Austria Division of Oncology, Medical University of Graz, Graz, Austria; 12 Department of Urology, Medical University of Graz, Graz, Austria; 13 Department of Oncology, Military Institute of Medicine, Warsaw, Poland; 14 Discovery Sciences, BioPharmaceuticals R&D, AstraZeneca, Cambridge, CB2 0AA UK; 15 Department of Oncology, University of Cambridge, Cambridge, CB2 0QQ UK; 16 Hutchison/MRC Research Centre, University of Cambridge, Cambridge, CB2 0QQ UK; 17 Christian Doppler Laboratory for Liquid Biopsies for Early Detection of Cancer, Graz, Austria

**Keywords:** Renal cancer, Cell-free tumor DNA (ctDNA), Personalized analysis, Predictive biomarker, Heterogeneity

## Abstract

**Background:**

Cell-free tumor-derived DNA (ctDNA) allows non-invasive monitoring of cancers, but its utility in renal cell cancer (RCC) has not been established.

**Methods:**

Here, a combination of untargeted and targeted sequencing methods, applied to two independent cohorts of patients (*n* = 91) with various renal tumor subtypes, were used to determine ctDNA content in plasma and urine.

**Results:**

Our data revealed lower plasma ctDNA levels in RCC relative to other cancers of similar size and stage, with untargeted detection in 27.5% of patients from both cohorts. A sensitive personalized approach, applied to plasma and urine from select patients (*n* = 22) improved detection to ~ 50%, including in patients with early-stage disease and even benign lesions. Detection in plasma, but not urine, was more frequent amongst patients with larger tumors and in those patients with venous tumor thrombus.

With data from one extensively characterized patient, we observed that plasma and, for the first time, urine ctDNA may better represent tumor heterogeneity than a single tissue biopsy. Furthermore, in a subset of patients (*n* = 16), longitudinal sampling revealed that ctDNA can track disease course and may pre-empt radiological identification of minimal residual disease or disease progression on systemic therapy. Additional datasets will be required to validate these findings.

**Conclusions:**

These data highlight RCC as a ctDNA-low malignancy. The biological reasons for this are yet to be determined. Nonetheless, our findings indicate potential clinical utility in the management of patients with renal tumors, provided improvement in isolation and detection approaches.

**Electronic supplementary material:**

The online version of this article (10.1186/s13073-020-00723-8) contains supplementary material, which is available to authorized users.

## Background

Renal cell carcinoma (RCC) is the most lethal urological malignancy with 50% of patients that develop the disease dying from it [1]. Clinical management challenges include the following: early diagnosis; differentiation of histological subtypes, i.e., chromophobe RCC (chRCC) from clear cell RCC (ccRCC) or benign oncocytoma; identification of patients with minimal residual disease following intended curative nephrectomy which will allow improved stratification of patients for adjuvant therapy trials; and predicting and tracking of response to targeted therapies. RCC has well-established pathological and genetic heterogeneity [2], which confounds development of personalized medicine [3]. Moreover, ccRCC exhibits a broad range of metastatic phenotypes [4] highlighting the need for longitudinal sampling. Due to the invasive nature of the procedure and failure to capture genetic heterogeneity, tissue biopsies potentially inadequately inform treatment decisions [5]. A “liquid biopsy,” providing an admixture of the entire tumor burden of a patient, may offer a non-invasive alternative to traditional tumor sampling techniques. Cell-free DNA (cfDNA), which in patients with cancer contains cell-free tumor-derived DNA (ctDNA), represent one such promising liquid biopsy strategy [6–8].

Despite showing great promise in various cancers [6], there is little and often contradictory data of ctDNA as a tool in RCC in locally advanced and metastatic RCC [6, 9, 10]. As such, there remains an unmet need for the characterization of the levels, and potential clinical utility, of ctDNA in renal cancers of differing stage and subtype [11, 12]. Furthermore, while evidence suggests ctDNA in the urine can be informative in urological cancers [13, 14], no previous study has assessed the presence of ctDNA in the urine of RCC patients. Here we aimed to determine the presence, levels, heterogeneity, and potential clinical applications of ctDNA in plasma and urine of 91 patients with renal tumors ranging from benign oncocytomas through to metastatic RCCs using both untargeted genome-wide and targeted sequencing approaches.

## Methods

### DIAMOND study sample collection

**Fig. 1:**
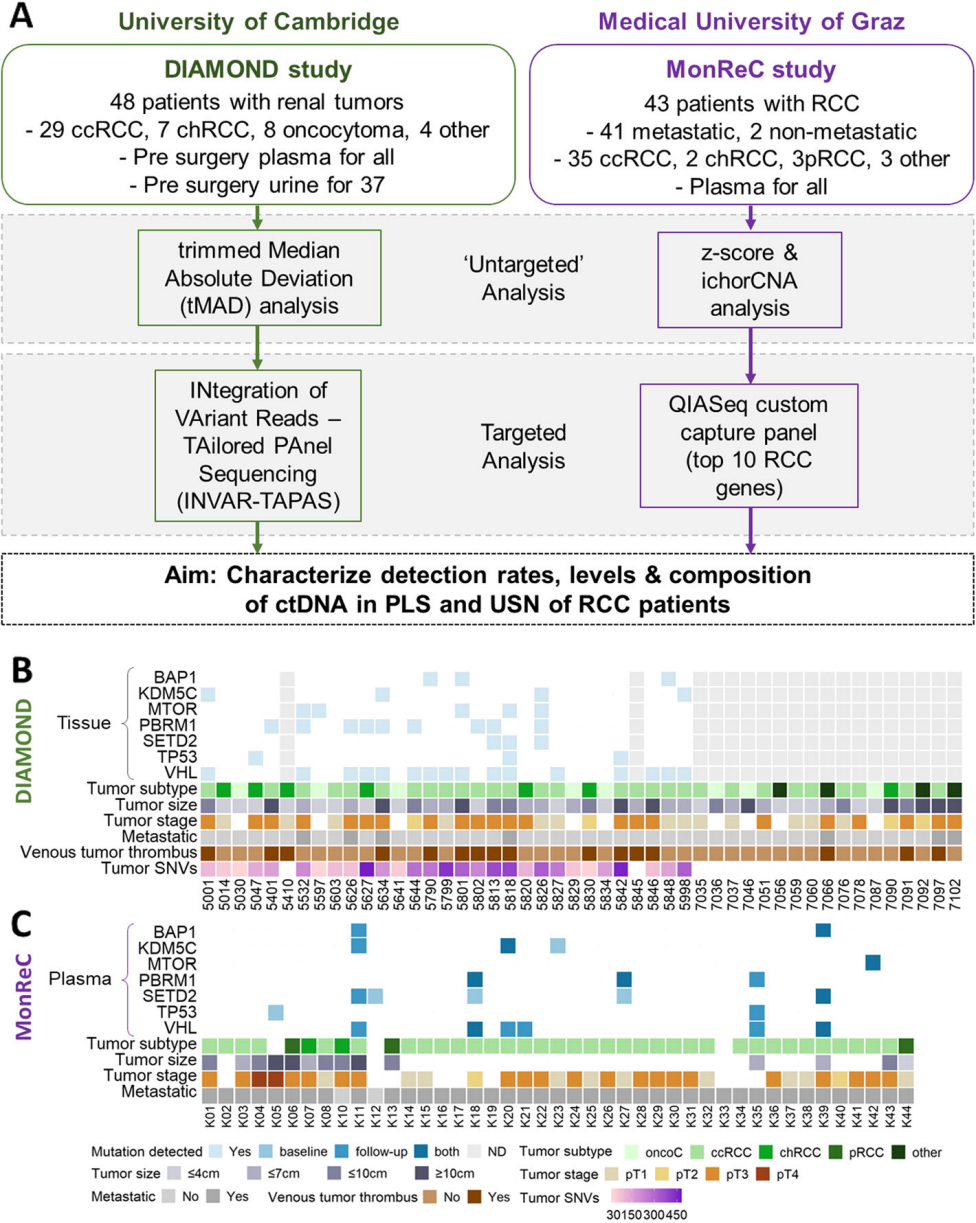
Study design, patient characteristics, and tumor genomic profile. **a** ctDNA analysis in RCC patients was applied to two patient cohorts, DIAMOND and MonReC. Initially, untargeted sequencing methods were applied to samples. For DIAMOND, tMAD analysis of sWGS data was applied. For MonReC, a combination of z-score analyses of mFAST-SeqS data and ichorCNA analysis of sWGS data was applied. Subsequently, targeted sequencing methods were used. For DIAMOND, INVAR-TAPAS was applied to patient plasma (*n* = 29) and urine (*n* = 20). For MonReC, a QIASeq custom capture panel targeting the 10 most commonly mutated genes in RCC patients was applied. **b** For DIAMOND, plasma (*n* = 48) and urine (*n* = 37) were collected from patients with a range of tumor subtypes and stages. Specifically, 29 ccRCCs (11/1/16 stage I, II, and III respectively), 7 chRCCs (2/2/3 stage I, II, and III), 8 oncocytomas, 1 patient with papillary RCC (stage III), 1 patient with a MiT family translocation RCC (stage II), and 2 patients with oncocytic renal neoplasm. Shown, in descending order, are tumor tissue mutation status of frequently mutated RCC genes (pale blue cubes indicate that a mutation was detected, white space indicates that no mutation was detected, gray columns indicate that tissue was not available for that patient), tumor subtype, tumor size, tumor stage, metastatic at baseline, evidence of venous tumor thrombus, and number of tumor SNVs (targeted for INVAR-TAPAS). **c** For MonReC, plasma (*n* = 43) was collected from 41 patients with metastatic RCC and two with localized RCC. Shown, in descending order, are plasma mutation status (after QIASeq, blue, medium blue and dark blue cubes indicate that a mutation was detected at baseline, during follow-up, or at both time points respectively) of frequently mutated RCC genes, tumor subtype, tumor size, and metastatic at time of sampling. More comprehensive versions of **b** and **c** are provided in Additional file 1: Fig. S15

Patients with a range of renal malignancies were recruited to the DIAMOND study according to the local ethical guidelines (REC ID 03/018). Patient characteristics and renal tumor pathological details are presented in Fig. 1a, b and Additional file 1: Table S1.

Patients underwent partial or total nephrectomy as part of curative treatment or cytoreductive surgery. Tumor tissue, from 29 patients, was obtained during these procedures and samples were stored as either fresh frozen (FF) or formalin fixed paraffin-embedded (FFPE) specimens. An average of four spatially separate tumor regions per patient (range 2 to 10 regions, 128 across all patients) were obtained, in order to study and overcome tumor heterogeneity prevalent in renal cancer [2].

For FF samples, a small piece of tissue weighing < 20 mg was removed and DNA was extracted using a DNeasy Blood & Tissue kit (QIAGEN) according to the manufacturer’s protocol. For the FFPE samples, 2-mm-diameter and 3-mm^3^-deep cores were obtained and DNA was extracted using the GeneRead DNA FFPE kit (QIAGEN) according to the manufacturer’s protocol, apart from the 56 °C incubation step which was carried out overnight instead of for 1 h. This protocol utilizes uracil-*N*-glycosylases enzymes in order to remove artifacts resulting from the deamination of cytosine during the fixation process. All extracted DNA was quantified using the Qubit assay run on the PheraStar FSX platform (BMG LabTech).

From all DIAMOND patients, we collected blood plasma prior to surgery (mean 5.0, range 0–35 days pre-surgery) and samples were processed as follows. For samples collected prior to April 2016 (21/32 patients), 8 ml of blood was collected into EDTA tubes and, within 1 h, centrifuged at room temperature at 4000 rpm for 20 min. The plasma layer was subsequently decanted into separate cryotubes. The buffy coat layer was transferred into a sterile 2-ml microfuge tube for parallel use. For samples collected after April 2016 (11/32 patients), 12 ml of blood was collected into EDTA tubes and centrifuged at room temperature at 1600*g* for 10 min within an hour of collection. Avoiding the buffy coat layer, 4 ml of plasma was transferred to RNase-free microfuge tubes and spun on a bench top centrifuge at 13,300 rpm for 10 min. The supernatant was transferred to a 2-ml sterile microfuge tube and the pellet was discarded. The buffy coat layer was transferred from the original collection tube into a sterile 2-ml microfuge tube for parallel use. Once processed, all samples were stored at − 80 °C.

For 37 patients, urine samples were also collected prior to surgery (mean 8.9, range 0–35 days pre-surgery). For 15 patients, we isolated only urine supernatant (USN) while for the 22 remaining samples, we isolated both USN and urine cell pellet (UCP), as follows. From each patient, 30–50 ml urine was collected in a 50-ml falcon tube, and 0.5 M EDTA was added within an hour of collection (pH 8.0; 600 μl for 30 ml, final concentration 10 mM. For larger volumes of urine, the volume of EDTA was adjusted accordingly). After gentle inversion, the sample was spun at 2400*g* for 10 min. Subsequently, ~ 3.6 ml of supernatant was transferred into a separate cryotube. For UCP collection, an additional 1 ml of supernatant was transferred to a separate microfuge tube, while the remaining supernatant was discarded. The 1 ml supernatant was then returned to the original falcon tube containing the UCP. This was agitated and the remaining liquid was transferred to a sterile 2-ml microfuge tube. This was spun at 13,300 rpm for 10 min and the supernatant was discarded leaving a dry UCP for storage at − 80 °C.

As well as pre-surgery plasma and urine, from a subset of patients we also collected post-surgery plasma and urine (Additional file 1: Fig. S1). Furthermore, in addition to renal cancer patient samples, we obtained plasma (Sera Labs) and urine DNA (local collection) from healthy individuals to act as controls for mutation analysis.

DNA was extracted from the fluid samples, as well as matched buffy coat samples, using the QIAsymphony platform (QIAGEN). DNA was quantified using the Qubit assay on the PheraStar FSX plate reader and by digital PCR using probes targeting the *RPP30* gene. All patient and sample details are summarized in Fig. 1b and Additional file 1: Table S1.

### MonReC study sample collection

An independent cohort of patients was recruited to the Graz based MonReC (monitoring renal cancer) study (approved by Ethics Committee of the Medical University of Graz, Austria, approval number 27-210 ex 14/15 and by the Ethics Committee of the Military Institute of Medicine, Warsaw, Poland, approval number 33/WIM/2015). Written informed consent was obtained from all patients before blood draw.

For the MonReC study, plasma was obtained at first diagnosis of metastases, during several lines of treatment, and/or at every further instance of progression/development of new metastases along with the introduction of a new line of treatment. Patient details are summarized in Fig. 1c and in Additional file 1: Table S2.

We obtained 49 blood samples from 18 patients (mean age 62.5 years; range 46–81) from the Department of Urology and from the Division of Oncology, Department of Internal Medicine, at the Medical University of Graz, Austria. In addition, 204 plasma samples were collected from 25 patients with metastatic disease (mean age 58.9 years; range 41–68), recruited from the Department of Oncology at Military Institute of Medicine, Poland.

For the Graz cohort, 9 ml blood was drawn into EDTA-containing tubes containing 10% NBF (BD Biosciences) or Streck tubes. Blood drawn at the Medical University of Graz, Austria (18/43 patients), was immediately sent to the Institute of Human Genetics. Plasma was extracted as described previously [15] and stored at − 80 °C prior to analysis. For samples collected at the Military Institute of Medicine, Poland (25/43 patients), plasma was extracted there and stored at − 80 °C before shipping to Graz. cfDNA was extracted from 2 ml of plasma using QIAamp Circulating Nucleic Acid Kit (QIAGEN) according to the manufacturer’s protocol. DNA was quantified using Qubit dsDNA HS Assay Kit (Thermo Fisher Scientific).

### Library preparation and exome capture of tissue and germline samples from DIAMOND patients

In order to identify patient specific somatic mutations, whole-exome sequencing (WES) of all tumor tissue and germline buffy coat DNA samples was carried out. Fifty nanograms of DNA was fragmented by acoustic shearing (Covaris) according to the manufacturer’s instructions. Libraries were prepared using the Thruplex DNA-Seq protocol (Rubicon Genomics) using 5x cycles of PCR. Exome capture was performed using the TruSeq Exome Capture protocol (Illumina) with the addition of i5 and i7 specific blockers (IDT) during the hybridization steps to prevent adaptor “daisy chaining.” After capture, 8x cycles of PCR were performed. Libraries, before and after hybrid capture, were quantified using KAPA library quantification kits (KAPA) and fragment size distributions were determined using a Bioanalyzer or Tapestation (Agilent). Sequencing was performed on a HiSeq 4000 (Illumina).

### DIAMOND shallow whole-genome sequencing and trimmed median absolute deviation (tMAD) analysis

Shallow whole-genome sequencing (sWGS) was performed on all tissue, plasma, and urine (USN and UCP) samples. For each sample type, libraries were pooled in an equimolar fashion and 150 bp paired end sequencing was performed (to give an average 16.4 million reads per sample) using an Illumina HiSeq 4000.

For sWGS analysis, sequence data was analyzed using an “in-house” pipeline that consists of the following; paired-end sequence reads were aligned to the human reference genome (GRCh37) using BWA (version 0.7.13) [16] after removing any contaminant adapter sequences. SAMtools (version 1.3.1) [17] was used to convert files to BAM format. PCR and optical duplicates were marked using Picard-Tools’ (version 2.2.4) “MarkDuplicates” feature and these were excluded from downstream analysis along with reads of low mapping quality and supplementary alignments.

CNA calling of tissue was performed in R using the QDNAseq pipeline [18]. Briefly, sequence reads were allocated into equally sized (here 1 Mb and 50 kb) non-overlapping bins throughout the length of the genome. Read counts in each bin were corrected to account for sequence GC content and mappability, and bins corresponding to previously “blacklisted” (ENCODE) and manually blacklisted regions were excluded from downstream analysis.

For all sWGS data, we calculated the trimmed median absolute deviation (tMAD) from the copy number neutral state. This method is described in detail in Mouliere et al. [19]. Briefly, this method compares normalized read counts across genomic bins in cases against those from a cohort of healthy control samples and the median absolute deviation from log_2_R = 0 of segmented bins is calculated. To define the detection threshold, we measured the tMAD score for sWGS data from 46 healthy individuals and took the maximal value (median = 0.01, range 0.004–0.015). The approach has a sensitivity of 0.3% [19] and details can be found at its github page (https://github.com/sdchandra/tMAD). We downsampled all plasma bam files to 10 million reads and carried out analysis using a bin size of 30 kbp. We used two forms of normalization—(1) normalization to a plasma sample from a cohort of healthy controls and (2) normalization to the samples’ own mean logR. All plasma and USN samples were analyzed by method 1. UCP samples were analyzed by method 2 as no matched healthy control samples were available for that sample type. For these UCP samples, ctDNA was detected if we observed a signal that deviated from the copy number neutral state.

In order to enrich for tumor-derived cfDNA fragments in plasma, we employed in silico selection of sequence reads within particular DNA fragment size ranges, an approach demonstrated to enrich for mutant signal in plasma [19]. Here, we downsampled sequence bam files to 2 million reads and carried out tMAD analysis using a bin size of 500 kbp. If, after size selection, sequence bam files had < 2 million reads, they were considered as ineligible for tMAD analysis. Signal was normalized against a similarly downsampled cohort of healthy controls.

### MonReC Modified Fast Aneuploidy Screening Test-Sequencing System (mFAST-SeqS)

In order to estimate the tumor fraction prior to more expensive genome-wide and/or high-resolution approaches, all samples collected in Graz were analyzed using the mFAST-SeqS method. Briefly, this approach is based on the selective amplification of LINE-1 sequences. LINE-1 amplicon libraries were generated from 0.5–1 ng of plasma derived DNA according to our previously published protocol [20]. Libraries were pooled equimolarily and sequenced on the Illumina NextSeq or MiSeq platform generating a minimum of 100,000 single-end reads for each sample. LINE-1 read counts per chromosome arm and on a genome-wide level were counted and compared to a set of healthy controls and deviations were reflected in *z*-scores [20].

### MonReC shallow whole-genome sequencing and ichorCNA analysis

For a subset of MonReC samples, sWGS was performed. To this end, shotgun libraries were generated from 5 to 10 ng of plasma DNA using the TruSeq DNA Nano Sample Preparation Kit (Illumina, San Diego, CA, USA) as previously described [15]. Libraries were quantified by qPCR with the quality checked using a Bioanalyzer DNA 7500 Kit (Agilent Technologies). Pooled libraries were sequenced on the Illumina NextSeq or MiSeq platform using the 2 x75bp paired-end mode. Additionally, data were analyzed with the previously published ichorCNA algorithm to calculate tumor fraction from ultra-low-pass whole-genome sequencing [21]. Due to the low tumor fractions, we applied an updated version of the algorithm (https://github.com/broadinstitute/ichorCNA/wiki/Parameter-tuning-and-settings). Moreover, in silico size selection was performed to enrich for tumor-derived fragments. As the lower limit of detection of ichorCNA was previously determined as a tumor fraction of 0.03, samples with tumor fractions below that threshold were considered as ctDNA negative. Due to the lower number of reads after size selection, the number of total reads was also downsampled to enable comparable profiles and to exclude false positive calls (see Supplementary material). For samples with less than 2 million reads after size selection, samples were only considered as positive when somatic copy number alterations (SCNA) frequently found in RCC according to the ProGenetix database were observed.

### DIAMOND mutations calling of tissue WES data

Mutation calling of WES data was performed as follows: Sequence data were aligned with BWA MEM v0.7.15 [22] to the GRCh37/hg19 human reference genome assembly that includes unlocalized and unplaced contigs, the rCRS mitochondrial sequence (AC:NC_012920), human herpesvirus 4 type 1 (AC:NC_007605), and the hs37d5 decoy sequence. Duplicate read pairs based on aligned positions of each end were marked using Picard v1.122 (http://broadinstitute.github.io/picard).

We focused our mutation calling efforts on regions of the genome that had depth > 20× in the matched germline buffy coat sequencing data. For this, we used the CallableLoci tool from GATK [17]. Somatic mutations were called using Mutect2 [23]. In addition to Mutect2’s filters, we applied additional filters described in Additional file 1: Table S3 [24].

We excluded SNVs that represented likely SNPs by virtue of their having a population allele frequency of above 0.02 in the 1000 Genomes project global database [25]. Furthermore, we excluded variants that had an AF > 0 in any normal adjacent tissue samples.

Despite these filters, we observed a large excess of C:G>A:T mutations in sequencing data from FFPE samples. We explored features of the artifact that could be used to distinguish it from true somatic C:G>A:T calls and found that it had a distinctive sequence context; specifically, it was often preceded by C or T and G or A respectively (i.e., [C/T] C>A and [G/A] G>T). As such we filtered out all C:G>A:T calls that had this sequence context. The resulting lists of patient-specific SNVs were used to guide the TAPAS panel design. WES revealed an average 297.36 unique somatic single nucleotide variants (SNV) per patient that passed our quality thresholds.

### DIAMOND Tailored Panel Sequencing (TAPAS)—custom capture panel design based on WES data

A 2.077-Mb (57,306 probes) personalized capture panel was designed based upon the somatic SNVs identified by WES of patient FF and FFPE tissue samples. Significant filtering of SNVs was required, as outlined above. The panel was designed using Agilent’s interactive online design tool, Sure Design (Agilent). The probe tiling parameters used were as follows; Tiling density = 1×, Masking = Most stringent, Boosting = Maximize performance, Extension into repeats = 20, Strand = sense.

As well as targeting the patient specific mutations identified by WES of patient tumor samples, the open reading frames of oncogenes and tumor suppressor genes previously implicated in renal tumorigenesis were tiled. These included the genes *VHL*, *PBRM1*, and *SETD2* [2]. The promoter region of *TERT* was also tiled [26]. The complete list of genes tiled by the panel is shown in Additional file 1: Table S4 while all genomic regions targeted for hybrid capture, as part of the INVAR-TAPAS panel, are provided in Additional file 2.

### DIAMOND fluid library preparation and hybrid capture

Libraries were generated from 15 to 30 ng plasma and USN derived DNA using Thruplex Tag-Seq kits (Rubicon Genomics). Tag-Seq libraries contain unique molecular indexes (UMIs) that make it possible to trace a sequence read back to the original DNA fragment that yielded it. After 8–11 PCR cycles (dependent on input), libraries were quantified using KAPA library quantification kits. UCP DNA was sheared using acoustic shearing (Covaris) and 15–30 ng were used for library preparation as above.

333.3 ng each of three libraries was pooled and the 1000 ng mix was used for hybrid capture using the custom SureSelect XTHS panel described above (Agilent). Hybridization was carried out with the addition of i5 and i7 specific blockers (IDT). Captured libraries were amplified using 13 cycles of PCR. Captured libraries were quantified using KAPA library quantification kits, and sequenced across multiple HiSeq4000 (paired end 150 bp) lanes such that at least 30 million sequencing reads were obtained for each sample in order to allow sufficient duplication for the proper use of UMIs.

### DIAMOND INVAR-TAPAS ctDNA detection

Aligned sequence reads were “collapsed” using UMI sequences incorporated during library preparation. The CONNOR tool (https://github.com/umich-brcf-bioinf/Connor/blob/master/doc/METHODS.rst) was used, with the following settings; minimum family size = 2, requires percent majority = 90%.

We applied our INtegration of VAriant Reads - TAilored PAnel Sequencing (INVAR-TAPAS) approach to the custom capture sequence data (Additional file 1: Fig. S2). Briefly, the INVAR algorithm [27] aggregates signal across hundreds to thousands of mutant loci identified by WES and targeted by the custom capture panel (described above). Error suppression, through the use of UMIs, and consideration of mutation sequence context, fragment length, and tumor mutant allele fraction is used to diminish background noise levels and enrich for ctDNA signal. This generates a significance level for each of the patient specific loci, which are combined into an aggregate likelihood function. Sequencing data from DNA of patients using non-matched mutation lists are used as negative controls for receiver operating characteristic curve analysis to select a likelihood threshold for ctDNA detection. A global ctDNA allele fraction is determined by taking a background-subtracted, depth-weighted mean allele fraction across the patient-specific loci in that sample.

The sensitivity of ctDNA detection depends on the total number of informative reads (IR; unique molecules aligning to patient specific mutant loci) covering tumor-mutated loci. Samples with < 20,000 IR will have limited ability to detect ctDNA. Few IR can be the results of too few patient-specific mutations detected in tissue (< 100 mutations). In these situations, detection to levels below 1.0 × 10^− 4^ mAF would require re-analysis using greater amounts of input DNA and/or re-design of the capture panel targeting a greater number of patient specific mutations. As such, those samples with too few IR were excluded as technical failures.

### MonReC QIASeq custom panel sequencing

For mutation profiling, a customized QIAseq Targeted DNA Panel (CDHS-11685Z-538; QIAGEN) was used. The panel enriches 10 genes that are frequently mutated in RCC: *BAP1*, *KDM5C*, *MET*, *MTOR*, *PBRM1*, *PIK3CA*, *PTEN*, *SETD2*, *TP53*, *VHL*. Libraries were prepared from 10 ng plasma DNA according to the QIAseq targeted DNA Panel Handbook (R2). Briefly, the DNA template was enzymatically fragmented, end-repaired, and A-tailed. Each DNA molecule was then tagged using an Illumina-specific adapter containing a UMI. Target regions were enriched by one target-specific primer and one universal primer. Finally, library amplification and completion of Illumina adapters was done in a universal PCR with 18 cycles. Libraries were quantified using the QIAseq Library Quant Assay (QIAGEN), and the fragment size was checked using the Bioanalyzer High Sensitivity DNA Kit (Agilent Technologies). Libraries were pooled equimolarily and sequenced on the Illumina NextSeq platform. On average, 3.47 million reads (range 1.33–5.7 M) were obtained per sample. Raw sequencing data generated by the QIAseq Targeted DNA Panel was analyzed using the QIAseq targeted DNA Panel Analysis pipeline, which processes the UMI information to distinguish between true variants and sequencing errors based on smCounter V1 [28]. All variants that did not pass the predefined quality criteria from smCounter were dismissed. Moreover, we filtered synonymous variants and variants present with minor allele frequencies of > 1% in population frequency databases (ExAC, gnomAS, 1000 g, TOPMED) were considered as polymorphisms. In order to increase variant calling stringency, we analyzed all samples in duplicate (median raw sequencing depth 9688.3, range 4176.7-17043.9), and only considered variants to be real if identified in both replicates. All detected variants were visually checked using Integrative Genomics Viewer (IGV) (version 2.3.58). Sensitivity assessment of two independent dilution series using SeraCare reference material revealed detection rates of 90% and 100% for 0.02 mAF, 70% for 0.01, and 25% for 5.0 × 10^− 3^ based on the evaluation of 10 different mutations. Variants with an expected mAF of 2.5 × 10^− 3^ and 1.3 × 10^− 3^ could not be detected.

### ctDNA detection and comparison against patient tumor size and presence of renal vein or inferior vena cava tumor thrombus

For DIAMOND patients, we assessed whether there was a difference in the distribution of tumor sizes amongst patients with detected ctDNA as compared to those in whom ctDNA was not detected. Tumor size was determined as the maximum diameter of the primary tumor on abdominal CT scan. We determined whether there was a relationship through the use of Mann-Whitney’s *U* test.

Similarly, we compared ctDNA detection in patients displaying evidence of extension of a tumor thrombus into the renal vein or inferior vena cava, as assessed on cross-sectional imaging, with those that did not have a tumor thrombus. Fisher’s exact test was used to determine associations between detection of ctDNA (in individual and combined fluids, by INVAR-TAPAS +/− tMAD) and tumor thrombus extension, with a significance threshold of *p* < 0.05.

In both cases, these were exploratory analyses, and to confirm the corresponding findings and hypotheses, further confirmatory tests are required.

### Ki-67 staining of DIAMOND tissue

We compared ctDNA detection with the Ki67 cellular proliferation rate in matched tumor cells, as levels of Ki67 have previously been found to correlate well with levels of ctDNA [29]. We carried out immunohistochemical (IHC) staining of FFPE tissue sections from a subset of DIAMOND patients, using an anti-Ki67 monoclonal antibody (MIB-1 clone at 1:100 dilution; DAKO Agilent Technologies LDA). The immunohistochemistry was scored manually at × 400 magnification. For each slide, 20 separate high-powered fields were assessed for positively stained tumor cell nuclei by a specialist uropathologist (AYW). Across the 20 regions, at least 6000 tumor cells were studied for each patient. An arbitrary proportion score was used to assess Ki67 levels with regions containing up to 0%, 1%, 10%, 30%, 75%, and 100% of positive cells being assigned a score of 0, 1, 2, 3, 4, and 5 respectively. The sum of these scores, across all 20 regions, was used as an indicator of the level of proliferation.

These values were subsequently compared between patients with detected and no-detected ctDNA with T-test *p* < 0.05 indicating a significant difference.

### Random forest model for ctDNA detection prediction

The model used here was based upon a classification model described in Mouliere et al. [19]. Briefly, the model considers the following fragmentation features (outlined in detail in the referenced manuscript) which were calculated from sWGS data: t-MAD, amplitude_10bp (the amplitude of 10 bp oscillations), P(20–150) (the proportion of fragments between 20 and 150 bp), P(160–180), P(20–150)/P(160–180), P(100–150), P(100–150)/P(163–169), P(180–220), P(250–320), P(20–150)/P(180–220). The model was trained using sWGS data from a cohort of “high ctDNA” cancer samples and was validated on “low ctDNA” cancer samples, including plasma from the sub-cohort of DIAMOND patients used in this study. Optimal classification of samples from cancer patients and controls was observed using a random forest (RF) machine-learning algorithm.

Here, we used the RF disease classification model to triage patient samples, predicting which RCC patients were likely to have sufficient ctDNA (in plasma or urine) for targeted sequencing by other, more sensitive, methods. We used “50% probability of cancer classification” as a threshold, comparing ctDNA detection amongst patients that fell above and below this value, as output by the RF model. We compared the ability of the model to triage patients using the INVAR-TAPAS method with or without tMAD in plasma alone, or in either fluid.

### Assessment of tumor heterogeneity and representation in plasma and urine

We assessed the heterogeneity of tumor samples from patient 5842, and its representation in plasma and urine samples obtained prior to nephrectomy. Mutations in tissue were called as described above. The SAMtools (version 1.3.1) [17] function mpileup was used to assess allelic content at mutant loci, with a mAF calculated for each site and this data was converted to a matrix. A heatmap was generated from this data using the R heatmap function. Hierarchical clustering was by mutations (columns) but not by sample (rows) according to Euclidean distance.

## Results

### Untargeted analysis of ctDNA in plasma and urine from patients with renal tumors

**Fig. 2:**
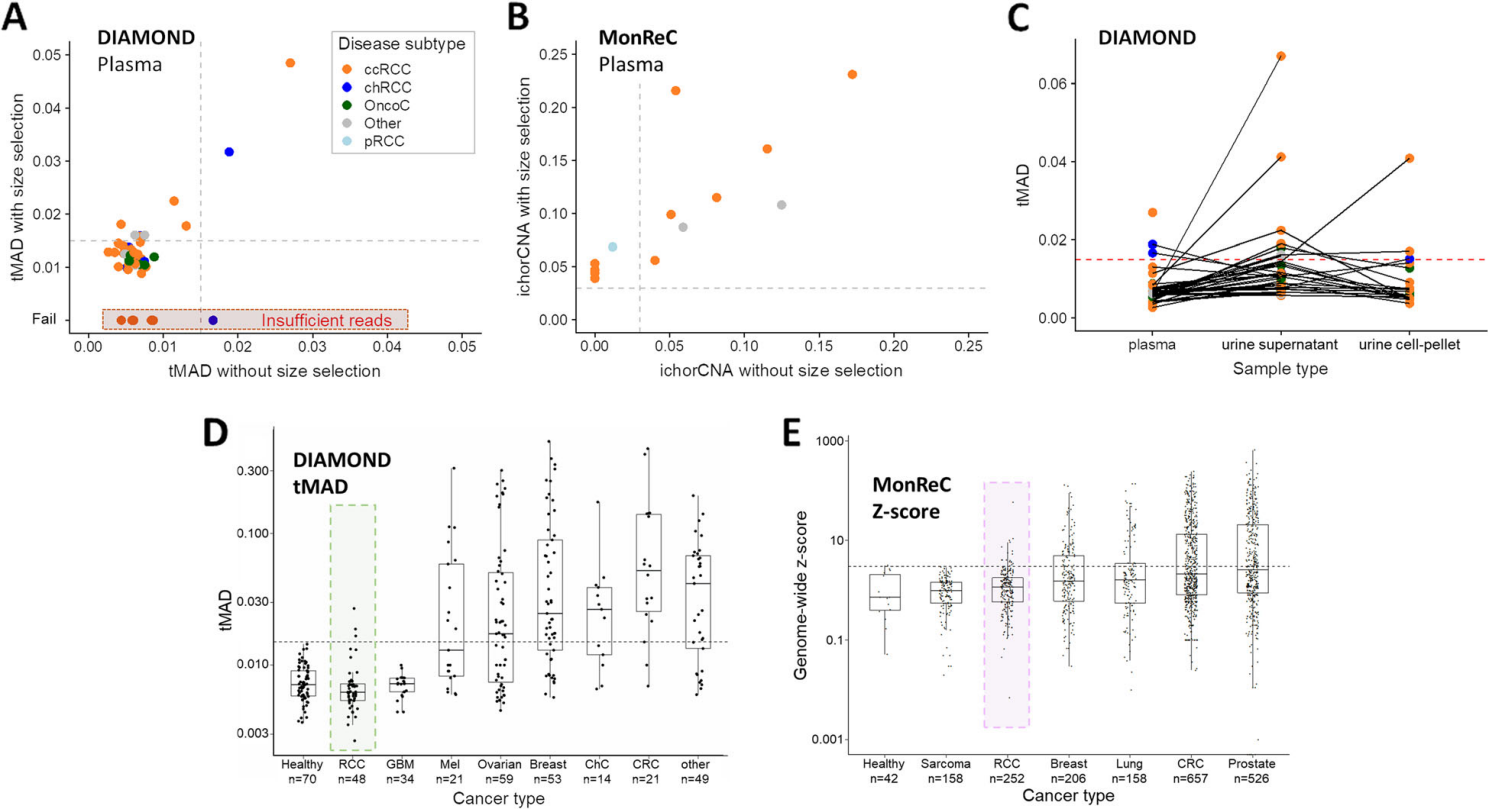
ctDNA detection using untargeted assays. **a** Distribution of tMAD scores across DIAMOND plasma samples (*x*-axis). Data points are colored according to disease subtype. A tMAD score of > 0.015 (gray dashed line) indicates SCNA, and thus ctDNA. ctDNA was detected in 3/48 (6.3%) plasma samples. Data on the *y*-axis show tMAD scores for the same plasma samples after in silico size selection for sequencing reads 90–150 bp in length. On average, the tMAD score increased 2.2-fold (range 1.25–4.83) and led to ctDNA detection in 8 additional patient samples, resulting in ctDNA detection in 11/48 (22.9%) DIAMOND patients. Four patient samples had insufficient sequencing reads after size selection for tMAD analysis (red highlight). **b** Tumor fraction of MonReC ctDNA-positive plasma samples (*n* = 14), as calculated by ichorCNA, before and after in silico size selection for sequencing reads 90–150 bp in length. On average, tumor fraction increased 2.2-fold (range 0.9–5.7) and revealed six patients with detected ctDNA, in addition to the eight patient samples detected without size selection. **c** Plot showing distribution of tMAD scores across DIAMOND plasma (no size selection), urine supernatant (USN), and urine cell pellet (UCP) samples. Samples from the same patient are connected by gray lines. The detection threshold is indicated by a red dashed line. **d** tMAD and **e** z-score distribution of RCC samples were compared to samples from other cancer types collected at the University of Cambridge [19] and Medical University of Graz respectively. Renal samples are highlighted. GBM = glioblastoma, Mel = melanoma, ChC = cholangiocarcinoma, CRC = colorectal cancer. A similar comparison was carried out using the ichorCNA metric (Additional file 1: Fig. S7C)

We applied a combination of rapid and cost-effective untargeted approaches, albeit with limited sensitivity, to establish a first measure of ctDNA presence and levels in patients with benign through to metastatic disease (*n* = 91 patients from the DIAMOND and MonReC studies; patient characteristics are shown in Fig. 1a–c and Additional file 1: Table S1-S2). In DIAMOND, patient plasma and urine were collected prior to partial or radical nephrectomy. In MonReC, the majority of patients had plasma collected during treatment for recurrent disease (mean time between first blood draw and surgery, 48 months, range 0–269). First, we assessed overall ctDNA levels using the trimmed median absolute deviation (tMAD) score calculated from shallow whole-genome sequencing (sWGS) [19] of plasma from 48 DIAMOND patients (Fig. 1b). As this method is dependent on the presence of SCNA, we applied sWGS/tMAD to matched tumor tissue, available from 28 of 48 patients (58.3%). All had SCNA (Additional file 1: Fig. S3), suggesting that SCNA are a valid ctDNA target in patients. However, in plasma, we detected SCNA in only 3 of 48 (6.3%) patient samples (Fig. 2a, one patient with metastatic ccRCC and 2 with non-metastatic chRCCs).

Next, we employed in silico selection of sequence reads within particular DNA fragment size ranges, an approach demonstrated to enrich for mutant signal in plasma [19, 30]. After selection of reads between 90 and 150 bp, 41/48 plasma samples from DIAMOND met the criteria for evaluation by tMAD analysis (> 2 million reads) (Additional file 1: Fig. S4A). On average, tMAD scores increased 2.2-fold (range 1.25–4.83) and led to ctDNA detection in 8 additional patients (Fig. 2a) including a patient with an oncocytoma (Additional file 1: Fig. S5A). Thus, in total, we detected plasma ctDNA in 11/48 (22.9%) DIAMOND patients.

The MonReC cohort consisted primarily of metastatic patients (two non-metastatic), most with their primary tumor removed (35/43 patients) (Fig. 1c). Samples were initially analyzed with untargeted methods, mFAST-SeqS [20] and sWGS (ichorCNA tumor fraction) (Additional file 1: Table S5). A mFAST-SeqS z-score of ≥ 3 indicates tumor fractions of > 3–10% (depending on the number and amplitude of SCNA) [31]. At baseline, *z*-scores ranged from − 0.6–3.7 (median = 0.7) with only 2/43 patients (4.7%) surpassing the detection threshold. The ichorCNA algorithm [21] revealed six further patients with detected ctDNA (8/43 = 18.6%; Fig. 2b) with tumor fractions up to 0.17 (median 0.07, range 0.04–0.17). As above, in silico size selection further improved the ichorCNA detection rate to 14/43 (32.6%) (Fig. 2b, Additional file 1: Fig. S6 and 7). On average, tumor fraction from ctDNA positive samples increased 2.2-fold (range 0.9–5.7) to a median of 0.08 (range 0.04–0.23). To assess the impact of size selection and the accompanying decrease of signal-to-noise ratio, we randomly downsampled the initial sequence data to a read count equivalent to that attained after size selection, and also analyzed a set of healthy controls. Despite finding that the proportion of small fragments (< 90–150 bp) in controls was much lower than in RCC samples (3.8% versus 7.8% of total reads) which in-turn led to a higher background, RCC-specific SCNA such as loss of 3p or 8p and gain of 3q or 8q were observed in none of the control samples.

As sampling of biofluids collected in close proximity to the tumor site may improve detection [14, 32], we analyzed urine supernatant (USN) samples from 37 DIAMOND cohort patients. Applying tMAD to USN for the first time (Additional file 1: Fig. S4B), ctDNA was detected in 8 patients (21.6%) (Fig. 2c, Additional file 1: Fig. S4C), including six patients with ccRCC, a patient with oncocytoma (not detected in plasma, Additional file 1: Fig. S5B), and a patient with MiT family translocation RCC. In addition to USN, we had access to urine cell pellet (UCP) DNA for 21 patients. UCP DNA is not cell-free but allows non-invasive detection of tumor DNA [14, 33]. UCP tMAD analysis revealed 3/20 (15%) patients with detected ctDNA, including one with localized ccRCC, the largest tumor of the cohort with a diameter of 23 cm. Comparison of plasma, USN and UCP data did not reveal a clear relationship in detection amongst these compartments (Fig. 2c, Additional file 1: Fig. S4C), confirming previous observations in bladder cancer [14, 34]. Considering only those patients for which we had access to plasma and urine, the detection rate increased to 16/37 (43.2%). Of note, two patients (including 5842 and 7092, Additional file 1: Table S1) had detected ctDNA in both plasma and urine.

Overall, untargeted sequencing methods employed here suggested low ctDNA detection rates in plasma and/or urine of patients with renal tumors (19/48, 39.6% in DIAMOND, 14/43, 33% in MonReC). Even in metastatic disease, the detection rate was only 35.4% (MonReC 13/41, DIAMOND 4/7). Comparison of plasma ctDNA levels, as quantified by tMAD, mFAST-SeqS, and ichorCNA, against other cancer types confirmed that ctDNA levels are lower in renal tumors (Fig. 2d, e, Additional file 1: Fig. S7C). Parallel analysis of urine (USN and UCP) also revealed low detection rates. ctDNA was detected in either fluid in patients with a range of tumor subtypes including, unexpectedly, patients with benign oncocytoma. Given these low detection rates, we hypothesized that techniques with greater sensitivity were required to quantify ctDNA in renal tumors.

### Targeted analysis of ctDNA yields improved detection rates

For DIAMOND patients, INtegration of VAriant Reads – TAilored PAnel Sequencing (INVAR-TAPAS) was used, an approach demonstrated to detect plasma ctDNA to parts per million [27] (Additional file 1: Fig. S2). This method relies on a priori knowledge of tumor specific mutations and thus, for 29 DIAMOND patients, we carried out whole-exome sequencing (WES) of matched tumor tissue and buffy coat (Additional file 1: Fig. S8). We observed extensive disease heterogeneity as previously described [2, 4] (Additional file 1: Fig. S9). Patient-specific mutations of key RCC genes are listed in Additional file 1: Table S6. A personalized capture panel was designed targeting all patient-specific SNV from tissue WES as well as the coding regions of 109 genes commonly mutated in renal tumors (Additional file 1: Table S4). Based on the requirements of the INVAR algorithm, seven plasma samples (7/29, 24.1%) had insufficient reads, resulting in limited ability to detect ctDNA, and were thus excluded as technical failures (Additional file 1: Fig. S10A).

**Fig. 3:**
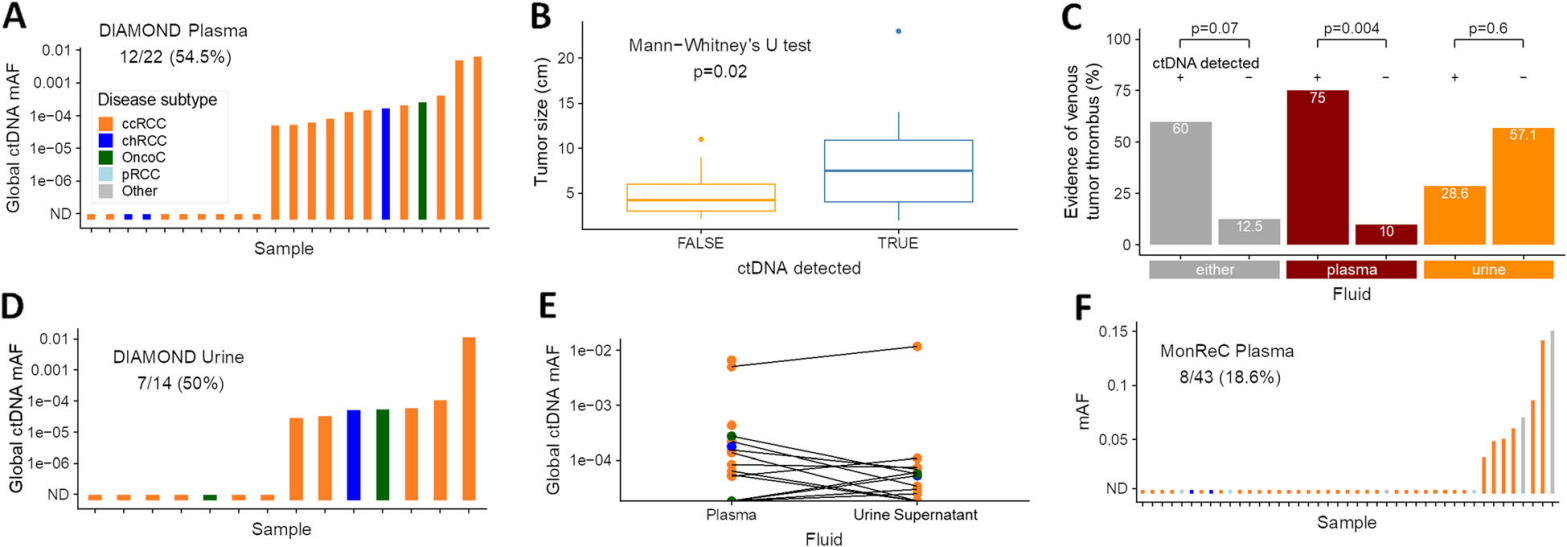
ctDNA detection using targeted assays. **a** Application of INVAR-TAPAS to DIAMOND plasma samples. ctDNA was detected in plasma of 12/22 (54.5%) patients, with global ctDNA mAF (gmAF) shown on the *y*-axis. Disease subtype is indicated by bar color, see insert for figure legend. **b** Assessment of the correlation between primary tumor size (diameter, cm), and ctDNA detection. Detection was via tMAD and/or INVAR-TAPAS, and in either fluid. This observation was driven by plasma (Additional file 1: Fig. S11A) with no apparent relationship in urine (Additional file 1: Fig. S11B). **c** ctDNA detection in plasma by INVAR-TAPAS was significantly more frequent amongst patients with venous tumor thrombus as compared to those without. This was not the case when considering ctDNA in urine or ctDNA in either fluid (Additional file 1: Fig. S12A-C). **d** INVAR-TAPAS was applied to DIAMOND USN samples. ctDNA was detected in 7/14 (50%) patients. **e** Comparison of gmAF of plasma and USN samples. In patients for whom we had access to both fluids, lines connect data points (Spearman’s rho = 0.28, *p* = 0.3). **f** Summary of targeted sequence analysis using a 10-gene QIASeq panel. Mutations at baseline were detected in 8/43 (18.6%) MonReC plasma samples. The *y*-axis denotes mAF which ranged from 3.5 × 10^−2^–0.15 (if two or more mutations were detected, the mean was calculated)

In the 22 remaining plasma samples, ctDNA was detected in 12 (54.5%) (Fig. 3a). Ten of these (83.3%) were ccRCC samples, including three detected by tMAD analysis of sWGS data. It is noteworthy that 9 of these patients had the largest evaluable ccRCC tumors in the cohort (7.4–23 cm). However, ctDNA was also detected in a patient with a small (2.8 cm) ccRCC with global mutant allele fraction, gmAF = 6.4 × 10^− 5^. Of the remaining patients with detected ctDNA, one had a small chRCC (2 cm) with gmAF 1.8 × 10^− 4^, and the other had a benign oncocytoma (3.9 cm) with gmAF of 2.7 × 10^− 4^. Assessment of all tumor subtypes revealed a significant (*p* = 0.02, Mann-Whitney’s *U* test) correlation between ctDNA detection and tumor size (Fig. 3b, Additional file 1: Fig. S11A). Similarly, ctDNA detection was more likely in patients with locally advanced RCC denoted by renal vein or inferior vena cava tumor thrombus (*p* < 0.05 for detection by INVAR-TAPAS +/− tMAD, Fisher’s exact test; Fig. 3c, Additional file 1: Fig. S12A-C). Conversely, Ki-67 assessed cellular proliferation rate did not correlate with detection (Additional file 1: Fig. S12D-F).

For the first time, we applied INVAR-TAPAS to USN from 20 patients. As in plasma, six samples (30%) had insufficient sequence reads and were excluded as technical fails. We detected ctDNA in USN of 7/14 patient samples (50%) (Fig. 3d; ccRCC *n* = 5, chRCC *n* = 1, oncocytoma *n* = 1). Two of these patients had detected urine ctDNA by tMAD analysis, while four had detected ctDNA in plasma (by INVAR-TAPAS or tMAD). Of note, the oncocytoma patient (histology confirmed; Additional file 1: Fig. S13) had ctDNA detected in plasma (gmAF USN 5.7 × 10^− 5^ vs plasma 2.7 × 10^− 4^). In contrast to plasma, there was no correlation between USN ctDNA detection and lesion size across all patients (Additional file 1: Fig. S11B), venous tumor thrombus invasion (Fig. 1b and Fig. 3c, Additional file 1: Fig. S12A-C) or proliferation rate (Additional file 1: Fig. S12D-F). There was no correlation between the global ctDNA mAF in plasma and urine (Spearman’s rho = 0.28, *p* = 0.3; Fig. 3e), though in the majority of patients, levels proved too low for accurate quantification of tumor fraction.

For metastatic RCC patients recruited to MonReC, no tumor tissue was available and so a de novo mutation calling approach was applied to plasma DNA. A gene panel targeting ten significantly mutated genes in renal cancers (*BAP1*, *KDM5C*, *MET*, *MTOR*, *PBRM1*, *PIK3CA*, *PTEN*, *SETD2*, *TP53*, *VHL)* [35] was used with a maximal achievable sensitivity of 5 × 10^− 3^ mAF. Based on existing data, one would expect a somatic mutation to be present in > 80% of metastatic ccRCC patients in at least one of these genes [36]. However, ctDNA was detected in only 8/43 (18.6%) baseline samples (Fig. 3f, Additional file 1: Table S5, S7). mAF ranged from 3.5 × 10^− 2^–0.18 with an average of 8.3 × 10^− 2^. Except for patient K42 (Fig. 1c), SCNA were detected in all of these samples after size selection. *SETD2* was the most frequently mutated gene in the cohort with a mutation being observed in 4 of the 8 mutation-positive patients (50%). *KDM5C*, *PBRM1*, and *VHL* were the next most frequently mutated genes with mutations being observed in 2/8 (25%) in both instances (Fig. 1c). In four patients, two or more mutations were identified.

We sought to identify whether untargeted plasma sWGS data could predict which patients would be suitable for downstream INVAR-TAPAS analysis, by application of a random forest model that considers fragmentation features of cfDNA (Additional file 1: Fig. S14A) to classify patient samples as healthy vs cancerous [19]. The model predicted detection of ctDNA by INVAR-TAPAS in plasma and/or urine in 91.7% of DIAMOND patients with an RF model score of > 50% vs. 36.4% of patients with an output < 50% (*p* = 9.4 × 10–3 Fisher’s exact test) (Additional file 1: Fig. S14). This data highlights the potential of the use of cost-effective techniques to triage patient samples for subsequent analysis by more expensive and time-consuming methods. However, the numbers of patient samples were small and independent validation of these findings is required.

**Fig. 4:**
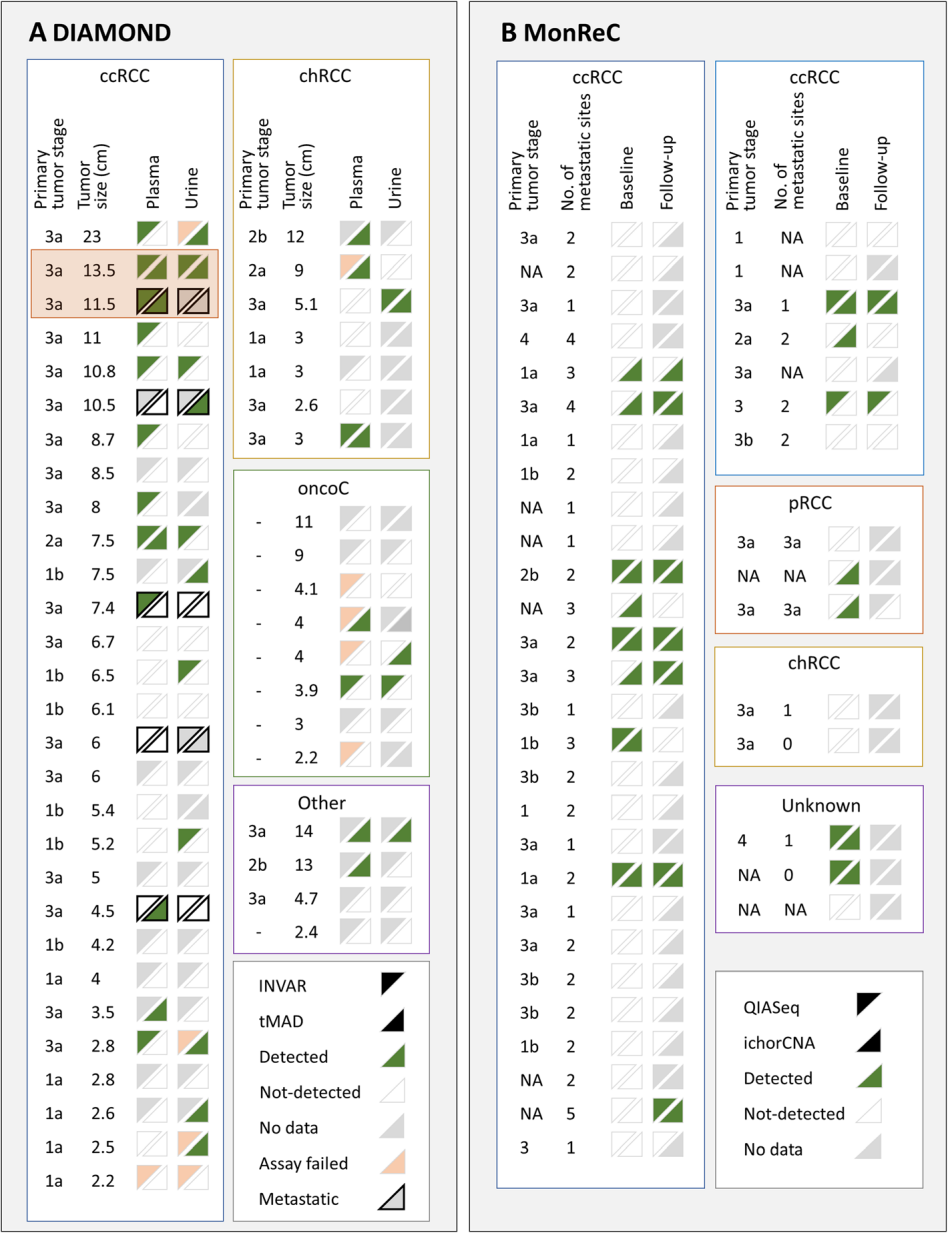
Summary of ctDNA detection in all patients and all biofluids. **a** Summary of ctDNA detection in baseline plasma (left of triangle box pair) and urine (right of pair) of DIAMOND ccRCC (left), chRCC (top right), and oncocytoma (oncoC, middle right) patients. Four patients with “other” disease subtypes are also shown (“other,” bottom right). Samples are ranked in descending order according to tumor size (cm). For each data point, the upper left triangle shows the results of INVAR-TAPAS analysis and the bottom right the results of tMAD analysis. Green triangles indicate samples in which ctDNA was detected, white triangles indicate samples in which ctDNA was not detected, gray triangles indicate no data available (because the assay was not applied to that sample, or no sample was available), and pink triangles indicate failed assay. Data points with a black outline indicate patients with metastatic disease at the time of sampling. DIAMOND patients 5842 and 5634 (longitudinal section) are highlighted with an orange box. **b** Summary of ctDNA detection in baseline (left of triangle box pair) and follow-up (right of pair) plasma of MonReC patients. Each subtype is shown in a separate box (ccRCC, clear cell; pRCC, papillary; chRCC, chromophobe; NA, unknown). The upper left triangle shows the results of QIASeq analysis, the bottom right the results of ichorCNA analysis. Triangle color, as above. Forty-one patients had metastatic disease and, where data was available, the number of metastatic sites is indicated. ctDNA detection are plotted alongside patient characteristics in Additional file 1: Fig. S15

ctDNA detection across all patient samples are summarized in Fig. 4 and Additional file 1: Fig. S15. Targeted analysis with a personalized approach (INVAR-TAPAS) improved ctDNA detection over untargeted analysis. Overall (targeted and untargeted data), 27 of 48 (56.3%) DIAMOND patients had detected ctDNA, whether in plasma or urine, though not all patient samples had the most sensitive approach applied to them or had both plasma and urine available. Of those that did, 11/13 (84.6%) patients had ctDNA detected. Of note, only one patient (5842) had ctDNA detected in both plasma and urine by both untargeted and targeted methods. For the MonReC cohort, the overall ctDNA detection rate of 34.5% was lower than for DIAMOND, most likely due to the use of less-sensitive de novo methods. Taken together, ctDNA detection in plasma and urine is challenging in patients with renal tumors, with tumor size (Fig. 3b) and renal vein or inferior vena cava tumor thrombus locally advanced disease (Fig. 3c) being the single greatest factors contributing to detection (as assessed in DIAMOND, Additional file 1: Fig. S11, S12A-C).

### Longitudinal analysis of ctDNA in renal tumors

**Fig. 5:**
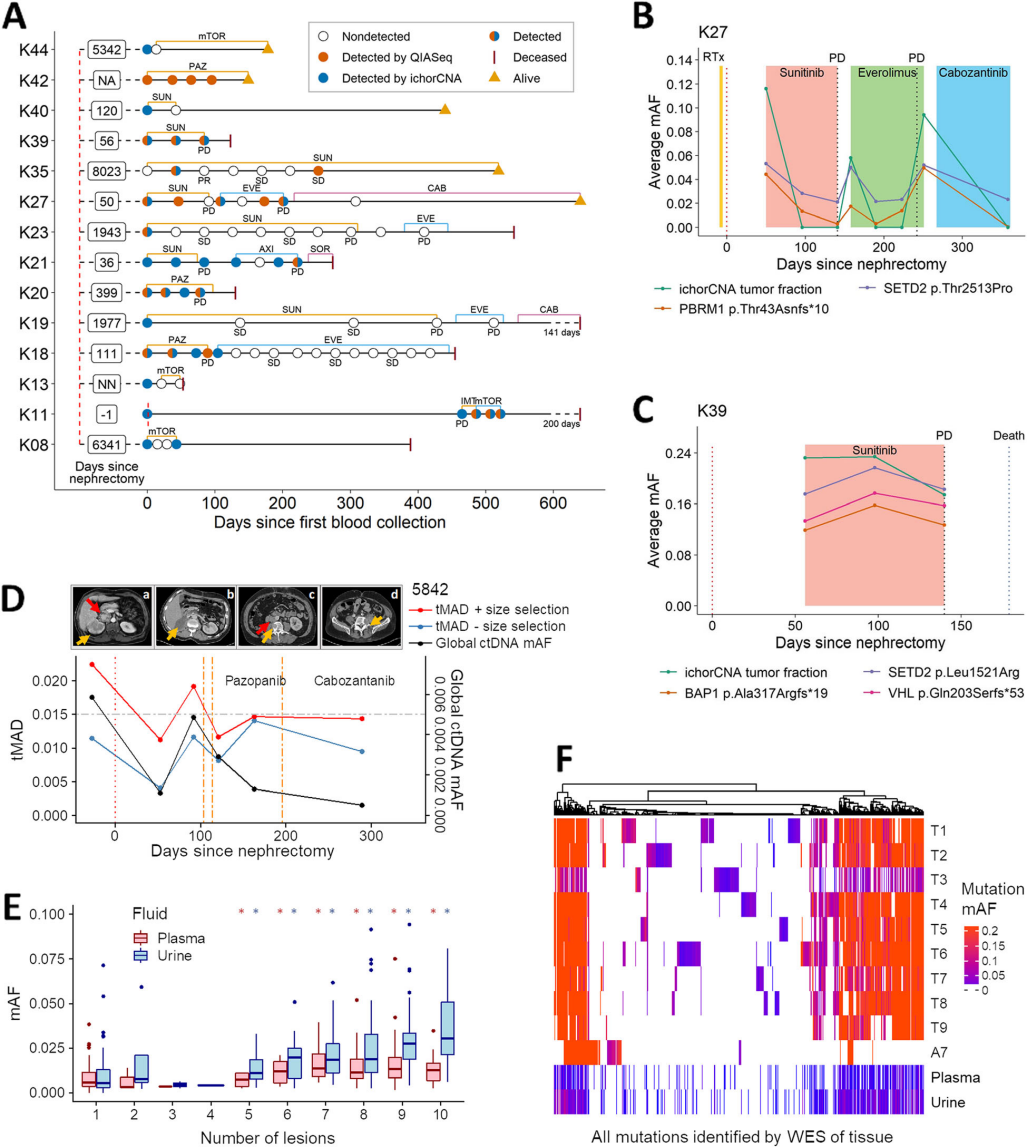
Longitudinal ctDNA analysis and assessment of intratumoral heterogeneity in plasma and urine. **a** Longitudinal cfDNA assessment of MonReC patients with metastatic RCC. Shown are disease courses of patients who had detected ctDNA with QIASeq and/or ichorCNA analysis. Time between nephrectomy and first blood draw is indicated in days (NA, not available; NN, no nephrectomy). Type and duration of treatment (mTOR = mTOR inhibitor; PAZ = pazopanib, SUN = sunitinib; EVE = everolimus; CAB = cabozantinib; AXI = axitinib; SOR = sorafenib; IMT = immune therapy) are indicated by colored lines. Most patients had detected ctDNA at progression (PD), whereas during stable disease (SD) or response (partial response, PR) ctDNA was undetected. **b**, **c** Plots demonstrating dynamic changes in ctDNA in longitudinal plasma from MonReC patients K27 and K39 respectively. Further details and patient specific plots are in Additional file 1: Fig. S17. **d** tMAD (left *y*-axis) and INVAR-TAPAS (right) analysis of plasma taken throughout the clinical course of DIAMOND patient 5842. Following nephrectomy (scan **a**; orange arrow = right renal tumor, red arrow = tumor thrombus), while INVAR-TAPAS global ctDNA levels (black line) drop, it remains detected (gmAF = 9.5 × 10–4) at day 53, indicating residual disease. Conversely, imaging did not detect residual disease at day 16 (**b**; normal renal fossa). ctDNA levels rise with disease spread, before falling again upon the commencement of radio- and chemotherapy. Of note, ctDNA levels continue to fall despite evidence of clinical progression. Further details are provided in Additional file 1: Fig. S18A-B. tMAD values before (blue line) and after (red line, gray circles = detected ctDNA) size selection are shown. Urine data are shown in Additional file 1: Fig. S18C. **e** Comparison of baseline ctDNA mAF in plasma (red) and USN (blue) and the number of tumor regions that mutation was observed in after multi-region sampling of 5842. *indicates significant difference as compared to mutations detected in just one region. **f** Heatmap of mutations detected across 10 tumor biopsies (T1–T9 = fresh frozen, A7 = FFPE) and baseline fluid samples from 5842, with vertical colored lines indicating individual SNVs. Hierarchical clustering was by mutation according to Euclidean distance. Color intensity corresponds to mutation mAF. While mutations show different representation in pre-surgery fluids (Additional file 1: Fig. S23), all mutation clusters, even those private to individual regions, are represented by at least one mutation in plasma and urine (Additional file 1: Fig. S24)

An aim of the MonReC study was to investigate the potential of plasma ctDNA to monitor treatment response in metastatic RCC, as such we had access to longitudinal plasma samples for 37/43 (86%) MonReC patients. During a median follow-up period of 6 months (range 0.4–19.2), serial plasma samples (median 5, range 2–21) were collected before and during treatment. mFAST-SeqS was used as an initial measure of tumor content [20, 37]. For all samples, the median genome-wide *z*-score was 1.0 (range − 0.9–58.0), and an elevated *z*-score was observed in 19/252 samples (7.5%) from 9 patients. In those samples, SCNA profiling revealed expected RCC aberrations, including 3p loss. Using a linear mixed model with a random intercept at the patient level, we found significant differences between baseline and treatment (Wald test, *p* < 0.001) and progression (Wald test, *p* = 0.0294) (Additional file 1: Fig. S16). Longitudinal mutation analysis was performed in 14 patients. Of those, 6 patients (K18, K20, K23, K27, K39, K42) had mutations called at baseline (Fig. 5a–c, Additional file 1: Fig. S17). Due to low detection rates at baseline, we analyzed additional samples whose collection coincided with clinical progression and identified three further patients (K11, K21, K35) with detected ctDNA. Moreover, ichorCNA was applied to available follow-up samples of 5 patients (K08, K13, K19, K40, K44) with detected ctDNA at baseline (Additional file 3: Table S8). For most patients, ctDNA levels assessed either by the QIASeq panel or ichorCNA were elevated at treatment initiation but decreased with response (Fig. 5b, Additional file 1: Fig. S17A-G). At progression, or when a treatment response was not gained, ctDNA increased or remained elevated (Fig. 5c, Additional file 1: Fig. S17A-G). For example, patient (K39) showed high levels of tumor DNA (> 0.1 mAF) at all available time points and died 2.8 months after the first blood draw, mirroring the rapidly progressing disease (Fig. 5c).

We obtained longitudinal plasma and urine from two DIAMOND patients (highlighted in Fig. 4 and Additional file 1: Fig. S1). Data are summarized in Additional file 1: Fig. S18–20. ctDNA levels largely fluctuated in accordance with clinical response as determined by standard imaging. Of note, the data from one patient highlighted that the detection of ctDNA (9.5 × 10^− 4^ gmAF) 53 days after radical nephrectomy pre-empted CT scan detection of minimal residual disease 82 days after surgery (Fig. 5d and Additional file 1: Fig. S1).

### Representation of tumor heterogeneity in plasma and urine

For all but two DIAMOND patients, the low ctDNA levels precluded meaningful assessment of representation of tumor heterogeneity in fluid samples. For DIAMOND patient 5842, we carried out WES of 10 spatially distinct tumor biopsies obtained after nephrectomy (Additional file 1: Fig. S21), identifying somatic mutations with varying apparent clonality. We compared the number of regions a mutation was called in, against the mAF of that mutation in plasma, urine, and tissue (Fig. 5e and Additional file 1: Fig. S22) and observed an incrementally rising mAF as more tumor regions were considered (Wilcoxon *T*-test *p* < 0.05). We assessed whether private mutations from each tumor region were represented in plasma and USN and found that both overcame this apparent heterogeneity with 90% and 100% of regions represented by at least one mutation in plasma and urine respectively (Fig. 5f and Additional file 1: Fig. S23). There was no evidence of one or more tumor regions having greater representation in fluids than others (Additional file 1: Fig. S24). These data confirm that plasma ctDNA can overcome tumor heterogeneity [38] and, for the first time, demonstrates that USN ctDNA is capable of the same (Fig. 5f). The ctDNA mAF varied between plasma and urine (mean mAF of detected mutations = 2.2 × 10^− 2^ vs 1.2 × 10^− 2^ respectively), with differing representation of likely driver genes including VHL (ENST00000256474.2:c.333_340+1delCTACCGAGG) (Additional file 1: Fig. S25). In patient 5634, plasma showed a similar ability to overcome heterogeneity with private clusters of mutations all represented in baseline plasma (Additional file 1: Fig. S26).

## Discussion

Here we present the most comprehensive assessment of ctDNA in renal tumor patients to date, using several state-of-the-art approaches applied to tissue and liquid biopsy samples from two independent, prospective clinical cohorts. These cohorts were complementary: (a) DIAMOND represented patients with the full range of renal tumors from benign to locally advanced and metastatic patients and (b) MonReC evaluated metastatic RCC patients treated with multiple systemic therapies and longitudinal follow-up/biosampling allowing the predictive ability of ctDNA to be assessed. We sought to take a bottom-up approach to determine if inexpensive, untargeted liquid biopsy approaches could be applied to RCC, as they have been successfully employed in breast [39], colorectal [40] and prostate cancer [41]. These methods enabled ctDNA detection in 30–40% of patients with RCC. Even in metastatic patients these methods achieved only moderate detection rates indicating generally low levels of tumor-derived DNA. Personalized high-resolution methods, which are more expensive, were used with incremental success.

RCC is often an aggressive, angiogenesis-driven malignancy, in a vascular organ with frequent cellular necrosis. As such, it is surprising that we observed such low ctDNA levels. Our data suggest that the probability of detecting ctDNA rises with increasing size of the primary tumor. Furthermore, amongst patients with locally advanced tumor growth, such as growth of a tumor thrombus into the renal vein or inferior vena cava, ctDNA detection in plasma, but not urine, was significantly more frequent. In contrast, tumor proliferation rate did not predict ctDNA detection as observed in patient-derived xenograft models [42] and lung cancer [29]. Surprisingly ctDNA detection was also limited to approximately a third of patients with metastatic disease from the MonReC cohort, albeit with substantially higher tumor fractions than observed in DIAMOND. An initial aim of this project was to determine the predictive ability of ctDNA in plasma and urine. Unfortunately, our ability to assess this was impacted by the low levels and detection rates of ctDNA in RCC. Nonetheless, we have attempted to correlate ctDNA levels with disease course that included several rounds of a variety of treatments.

There is little published data characterizing ctDNA levels in RCC. Initial studies suggested low detection rates and/or levels of ctDNA in locally advanced and metastatic RCC. More recently, targeted sequencing detected ctDNA in 30% of 53 RCC patients [10]. Conversely, Pal and colleagues detected ctDNA in 78.6% of 200 metastatic patients using the Guardant360 plasma assay (Guardant Health), though with a median of one genomic alteration per sample [43]. The same authors detected ctDNA in a further 18/34 (53%) metastatic RCC patients and, as echoed by our data, observed a possible correlation between detection and lesion diameter [44]. Likely reasons for the lower detection rate amongst metastatic patients in the MonReC cohort include our use of a smaller gene panel which has a detection limit of 5.0 × 10^− 3^ as compared to 2.0 × 10^− 4^ for Guardant360. Moreover, their study analyzed more than 70 RCC associated genes, including *EGFR*, *NF1*, and *ARID1A*, many of which were not included in our assay. Of note, neither of these two studies reported the range of detected mAFs, meaning that a direct comparison with our data was not possible. Nevertheless, the use of larger gene panels, or personalized assays such as INVAR-TAPAS, is likely to increase ctDNA detection rates. Considered with our data, it is clear that a consensus concerning ctDNA levels in RCC has yet to be reached.

For select DIAMOND patients, we also assessed tumor DNA content in urine (supernatant and cell-pellet) and found similar levels and detection rates with minimal overlap between fluids, with only seven patients having detected ctDNA in both plasma and urine. This data suggests that the mechanisms that determine the release and levels of ctDNA in plasma and urine of patients with renal tumors vary, a finding that requires further mechanistic analysis. In particular, it will be important to evaluate urine samples from patients with metastatic disease (of which we had only a single case in this study), to compare the representation of ctDNA in plasma vs. urine in patients without their primary disease in situ*.*

We further aimed to assess intratumoral heterogeneity through the comparison of multi-region sampled tumor tissue and ctDNA. Unfortunately, analysis of further patients was hampered by their low ctDNA levels. Nevertheless, analysis of two well characterized DIAMOND patients revealed that mutations from the majority of sampled tumor regions were detected in plasma. In addition, we show for the first time that genetic heterogeneity is represented in ctDNA from urine. As such, while limited, our data suggest that ctDNA analysis of both fluids has the potential to overcome intratumoral heterogeneity that is prevalent in renal cancers [2].

We also found that ctDNA can indicate minimal residual disease after nephrectomy (in one case pre-empting detection by imaging by 29 days), as well as disease progression on systemic therapies. Another noteworthy finding was the detection of ctDNA in the plasma and urine of patients with benign oncocytomas and early-stage ccRCCs. For the former, this is particularly surprising given the benign nature of these lesions. While differentiation of small renal masses into ccRCC, chRCC, or oncocytoma can be challenging using renal tumor biopsy, these data hint at the possibility of non-invasively differentiating small renal masses to guide decisions over invasive surgery versus active surveillance.

We recognize the limitations of our study. By including a broad range of renal mass patients, there was a limited number of patients with each disease stage. Furthermore, our pre-analytical knowledge evolved during recruitment to the DIAMOND cohort meaning not all sample types and time points were available for all patients. Indeed, due to the low detection rates, longitudinal monitoring was applicable in only a minority of patients. Moreover, different techniques were applied to different patient cohorts, though this in turn reinforces the general statement that ctDNA in RCC is challenging and further developments are needed for clinical utility. In future work, standardization of timing of blood draws especially in relation to systemic therapy dosing requires further study. Furthermore, larger studies of ctDNA in ccRCC are now required to better determine its clinical utility and validate any prognostic or predictive utility. Beyond this, assays that target multiple biomarkers, including proteins [45] and methylated cfDNA [46], will improve sensitivity for detecting and interpreting tumor signal. Multiple questions remain about this ctDNA-low malignancy but there is no doubt that further study is warranted and will inform approaches for other ctDNA-low tumor types.

## Conclusions

Our data highlight RCC as a ctDNA-low malignancy. However, we illustrate potential clinical utility for the management of patients with RCC. Further advances in pre-analytical and analytical processes, for both urine and plasma, are needed for this potential to be fulfilled.

## Supplementary information

Additional file 1: **Table S1.** Summary of patient characteristics of the DIAMOND cohort. **Table S2.** Summary of patient characteristics of the MonReC cohort. **Table S3.** Summary of filters applied to somatic single nucleotide variants (SNV) calls from Mutect2, for mutation calling of DIAMOND tissue samples. **Table S4.** Genes targeted for open reading frame sequencing. **Table S5.** Summary of samples with detected ctDNA at baseline of the MonReC cohort. **Table S6.** Mutations identified in tissue of in *VHL*, *PTEN*, *TP53*, *SETD2*, *PBRM1*, and *BAP1* of patients subsequently analyzed with INVAR-TAPAS (*n* = 29). **Table S7.** Mutations identified at baseline in the MonReC cohort using a QIASeq custom panel targeting 10 frequently mutated genes in RCC. **Fig. S1.** Clinical records of select DIAMOND patients. **Fig. S2.** Schematic explaining the INVAR-TAPAS approach. **Fig. S3.** Summary of SCNA observed in matched tumor tissue from select DIAMOND patients. **Fig. S4.** tMAD - interrogation of plasma and urine data. **Fig. S5.** Assessment of SCNA landscape of matched tumor tissue supports their classification as oncocytoma. **Fig. S6.** Improved detection of SCNA by in silico size selection. **Fig. S7.** Comparison of the distribution of ichorCNA tumor fraction and tMAD score at baseline, and assessment of ichorCNA scores between cancer types from the MonReC cohort. **Fig. S8.** Summary of tumor tissue WES of the DIAMOND patients. **Fig. S9.** Tumor heterogeneity in RCC. **Fig. 10.** Summary of global ctDNA levels relative to informative reads in INVAR-TAPAS data from patient plasma and urine. **Fig. S11.** Correlation of tumor size and ctDNA detection. **Fig. S12.** Correlation of venous tumor thrombus and cell proliferation rates with ctDNA detection. **Fig. S13.** Confirmation of pathological classification as oncocytoma. **Fig. S14.** Application of a fragmentation feature based random forest model to predict patients with detected ctDNA. **Fig. S15.** Summary of ctDNA detection in all patients and all biofluids. **Fig. S16.** Comparison of z-score distribution at baseline, during treatment and when progression occurred. **Fig. S17.** Longitudinal monitoring of mutations detected with the QIAseq custom panel and ichorCNA tumor fractions. **Fig. S18.** Comparison of imaging data and ctDNA levels (as predicted by INVAR.TAPAS and tMAD). **Fig. S19.** Comparison of SCNA landscape of matched tumor tissue, normal adjacent tissue, and longitudinal plasma samples from DIAMOND patient 5634. **Fig. S20.** Comparison of imaging data and ctDNA levels (as predicted by INVAR.TAPAS and tMAD). **Fig. S21.** Tumor map of patient 5842. **Fig. S22.** Comparison between mAF and mutation representation in tumor lesions. **Fig. S23.** Representation of private mutations in the baseline plasma and USN samples of patient 5842. **Fig. S24.** Representation of mutations private to tumor regions in plasma and urine. **Fig. S25.** Distribution of mAF of patient specific mutations in plasma and urine from patient 5842. **Fig. S26.** Assessment of tumor heterogeneity representation in plasma from patient 5634.

Additional file 2. Bed file showing all genomic regions targeted by INVAR-TAPAS of DIAMOND patients.

Additional file 3. Table S8. Summary of all mutation data of the MonReC cohort.

## Data Availability

The following sequencing raw data sets have been deposited at the European Genome-phenome Archive (EGA; http://www.ebi.ac.uk/ega/), which is hosted by the EBI, under the accession number EGAS00001003530: (1) sWGS data of DIAMOND cohort (tissue: EGAD00001005815, cfDNA from urine and plasma: EGAD00001005814), (2) sWGS data of the MonReC cohort (cfDNA from RCC patients: EGAD00001005804, non-cancer control: EGAD00001005805), (3) WES data of the DIAMOND cohort (EGAD00001005812), (4) INVAR-TAPAS data (mutation analysis of the DIAMOND cohort) (EGAD00001005813), (5) QIASeq data (mutation analysis of the MonReC cohort) (EGAD00001005806). mFAST-SeqS data from non-cancer controls are available under the accession number EGAS00001001133 (dataset EGAD00001001314) whereas RCC data of the MonReC cohort are available at https://figshare.com/ (dataset mFAST-SeqS from plasma DNA of RCC patients 10.6084/m9.figshare.11814681). The tMAD metric is included as part of the CNAclinic package, available at https://github.com/sdchandra/tMAD. IchorCNA algorithm to calculate tumor fraction from ultra-low-pass whole-genome sequencing is available at https://github.com/broadinstitute/ichorCNA/wiki/Parameter-tuning-and-settings. Picard v1.122 to mark PCR duplicates is available at http://broadinstitute.github.io/picard. CONNOR tool to collapse UMI sequences from IVAR-TAPAS is available at https://github.com/umich-brcf-bioinf/Connor/blob/master/doc/METHODS.rst. Details of the INVAR-TAPAS panel, including the list of genes targeted for open reading frame sequencing and the genomic regions targeted for hybrid capture, are provided in Additional file 1: Table S4 and Additional file 2 respectively.

## Abbreviations

ccRCC: Clear cell RCC
cfDNA: Cell-free DNA
chRCC: Chromophobe RCC
ctDNA: Cell-free tumor DNA
EDTA: Ethylenediaminetetraacetic acid
FF: Fresh frozen
FFPE: Formalin fixed paraffin embedded
gmAF: Global mAF
INVAR-TAPAS: INtegration of VAriant Reads - TAilored PAnel Sequencing
IR: Informative reads
mAF: Mutant allele fraction
mFAST-SeqS: Modified Fast Aneuploidy Screening Test-Sequencing System
OncoC: Oncocytoma
PCR: Polymerase chain reaction
pRCC: Papillary RCC
RCC: Renal cell carcinoma
RF: Random forest
SCNA: Somatic copy number alteration
SNV: Single nucleotide variant
sWGS: Shallow whole-genome sequencing
tMAD: Trimmed median absolute deviation
UCP: Urine cell pellet
UMI: Unique molecular index
USN: Urine supernatant
WES: Whole-exome sequencing
